# The crucial choice of reference genes: identification of miR-191-5p for normalization of miRNAs expression in bone marrow mesenchymal stromal cell and HS27a/HS5 cell lines

**DOI:** 10.1038/s41598-020-74685-7

**Published:** 2020-10-20

**Authors:** É. Costé, F. Rouleux-Bonnin

**Affiliations:** CNRS ERL7001 GICC Team LNOx, University of Tours, Tours, France

**Keywords:** Biological techniques, Cell biology

## Abstract

Bone marrow mesenchymal stromal cells (BM-MSCs) have a critical role in tissue regeneration and in the hematopoietic niche due to their differentiation and self-renewal capacities. These mechanisms are finely tuned partly by small non-coding microRNA implicated in post-transcriptional regulation. The easiest way to quantify them is RT-qPCR followed by normalization on validated reference genes (RGs). This study identified appropriate RG for normalization of miRNA expression in BM-MSCs and HS27a and HS5 cell lines in various conditions including normoxia, hypoxia, co-culture, as model for the hematopoietic niche and after induced differentiation as model for regenerative medicine. Six candidates, namely miR-16-5p, miR-34b-3p, miR-103a-3p, miR-191-5p, let-7a-5p and RNU6A were selected and their expression verified by RT-qPCR. Next, a ranking on stability of the RG candidates were performed with two algorithms geNorm and RefFinder and the optimal number of RGs needed to normalize was determined. Our results indicate miR-191-5p as the most stable miRNA in all conditions but also that RNU6a, usually used as RG is the less stable gene. This study demonstrates the interest of rigorously evaluating candidate miRNAs as reference genes and the importance of the normalization process to study the expression of miRNAs in BM-MSCs or derived cell lines.

## Introduction

Mesenchymal stromal cells (BM-MSCs) represent a heterogeneous population of cells that exhibit different cellular, molecular and functional properties. The most common source of adult resident BM-MSCs is the bone marrow, where stem cells are capable of unlimited self-renewal and have high multilineage differentiation potential including osteogenic, adipogenic, and chondrogenic ones under appropriate conditions^1^. The unique differentiation ability of BM-MSCs may contribute to tissue regeneration and wound healing, thus making them useful for regenerative medicine^2^. They are also critical for maintaining hematopoietic homeostasis by controlling the balance of hematopoietic stem cells between quiescence/proliferation and self-renewal/commitment in hypoxic condition. Allogenic primary MSCs are casually used to study these two processes, but human bone marrow stromal cell lines such as HS27a and HS5 are often used as a surrogate, to eliminate interindividual donor variability associated with BM-MSC primary cells^3^. These two cell lines were derived from the same healthy donor and are widely used to mimic hematopoietic microenvironment. However, these cells differ in function^4^. CD318^−^ CD146^+^ HS27a cells closely resemble the primary CD146^+^ cells and express stem cell niche-associated proteins such as SDF-1, angiotensin, osteopontin, and VCAM-, allowing them to establish a stem cell niche^5^. They also support cobblestone area formation whereas CD318^+^ CD146^−^ HS5 cells secrete growth factors (GM-CSF, G-CSF, IL-1, IL6, IL-8, IL11, MCP3, and MIP1a) enhancing the proliferation and differentiation of CD34^+^ cells^4,6^.

Increasing number of studies have been performed to improve the understanding of the molecular expression profile governing BM-MSCs features. Detailed explorations of the interactions between niche parameters and cellular or molecular components, such as normoxia versus hypoxia, co-culture or differentiation experiments, are a first step towards understanding tissue regeneration or bone marrow function^7,8^. All these processes are finely tuned partly by direct cellular interaction but also by paracrine secretions of BM-MSCs, which require strong gene expression control. microRNAs (miRNAs) have been implicated widely in the control of biological pathways including differentiation, proliferation and survival^9^. They are short non-coding RNAs of 18 to 26 nucleotides that mediate gene silencing by post-transcriptional regulation^10,11^. They account for about 1% of the human genome^12,13^. They are thought to be able to target at least 60% of human transcripts^14^ for which the miRNA/mRNA interaction lead to translation repression of mRNA targets or to degradation of mRNA molecules. Currently, there are 38,589 identified mature miRNA in the last release of miRbase (Release 22.1, October 2018; https://www.mirbase.org) and changes in their expression patterns regulate a kaleidoscope of physiological and pathophysiological pathways^15–19^. Thus, a deeper understanding of the complex networks of interactions coordinated by miRNAs would allow to design better therapeutic interventions. Therefore, expression profile regulation studies are essential. A key to performing a powerful miRNA expression analysis is to start off by an evaluation of the sample source, sampling methods, preservation and extraction. When comparing several studies, one must also assess the comparability of the methods used for expression analysis and the data normalisation strategies. Several techniques are available to quantify miRNA, the most used are Next Generation Sequencing (NGS), microarrays and quantitative polymerase chain reaction (qPCR). NGS is the only technique that allows the detection of non-identified miRNAs, but reveals to be both costly and heavy in terms of generated data^20^. Microarray is a simpler method than NGS which explain it notoriety, it allows to analyse a large set of genes at moderate costs, despite requiring large amounts of RNA material^21^. The qPCR is widely used, especially at the final step of the experimental strategy, to assert miRNA expression. This technique is the most sensitive and the cheapest, which explains its success as a routine assay. A major pitfall is the comparability between quantitative results of cell-associated miRNAs when cell culture conditions differ. However, reliable reference genes (RG) must be selected for data normalization when comparing expression level variations between different experiments. The selection and validation of these genes is a crucial step^22^ since there is no consensus as to which RGs to use for qPCR data normalization. The primary goal of this study was to validate endogenous miRNAs as RGs and not synthetic miRNAs in bone marrow mesenchymal stromal cells and in model cell lines. To do this, the cells were differentiated in the three lineages as performed in repair studies or cultured under normoxic/hypoxic conditions or co-cultured with hematopoietic cell lines in order to mimic niche conditions. We then selected 6 RGs commonly used in the published literature and studied their expression in all three conditions. The stability of these RGs was determined using geNorm, NormFinder and BestKeeper algorithms and by DeltaCt method. The outcome of the present study were validated on miR-21-5p reported to be expressed in BM-MSCs^23^.

## Results

**Figure 1:**
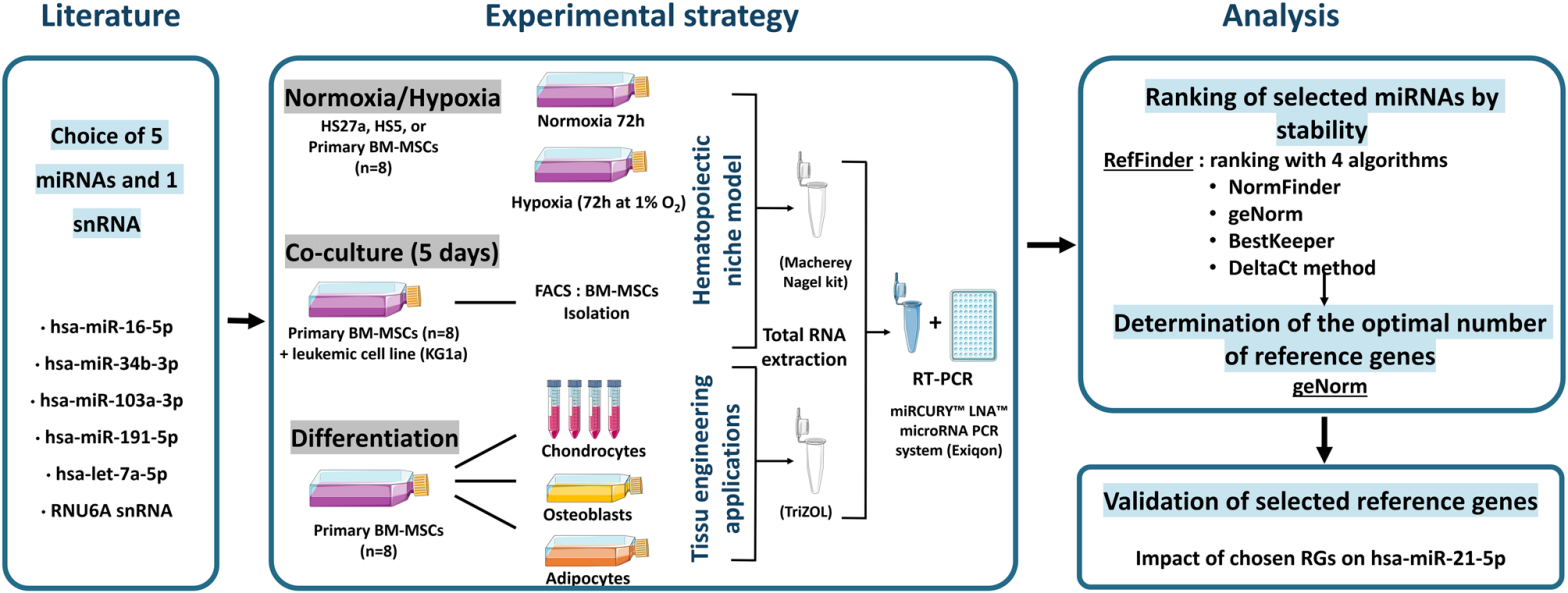
Strategy used for identification of reference genes. After selection of 6 RG candidates, BM-MSC were cultivated in different conditions: normoxia, hypoxia, co-culture and differentiation. After RNA extraction, the expression of candidates was analysed by RT-qPCR. Those results were analysed by geNorm and RefFinder to rank RG candidate by stability and geNorm was used to determine the optimal number of RGs. The selected RGs for each condition were next validated by studying the relative expression of miR-21-5p. Parts of the figure were drawn by using pictures from Servier Medical Art. Servier Medical Art by Servier is licensed under a CC BY 3.0 License (https://creativecommons.org/licenses/by/3.0/).

The whole study design is represented in the Fig. 1 and each step is developed in the results section.

### Quality control of experimental conditions

In order to verify that there is no bias in our experiments, we verified the differentiation state of BM-MSCs, the quality of RNA extraction protocols and the ability for a miRNA to be a candidate reference gene in qPCR analysis.

As shown in Supplemental Data 1, histological experiments verify the three differentiations ability of BM-MSCs (Supp Data 1).

Then extractions of total RNA were performed by using NucleoSpin miRNA kit (Macherey–Nagel) or by using TRIzol extraction. Supplementary Data 2 indicated that the Ct values are higher in TRIzol-extracted samples versus kit-extracted samples for BM-MSCs, HS27a and HS5 due to lower quality of extraction^24^. Moreover, the standard deviation (SD) of the mean of all 8 repetitions shown that Nucleospin extraction leads to higher reproducibility. Despite the superiority of Nucleospin miRNA kit in terms of reproducibility, the extracellular matrix produced by osteoblasts and the lipid vesicles inside adipocytes are not compatible with the column technology, thus forcing to use classical TRIzol extraction. On the other hand, the structure of chondrocytes aggregates requires a specific TRIzol extraction protocol.

**Figure 2:**
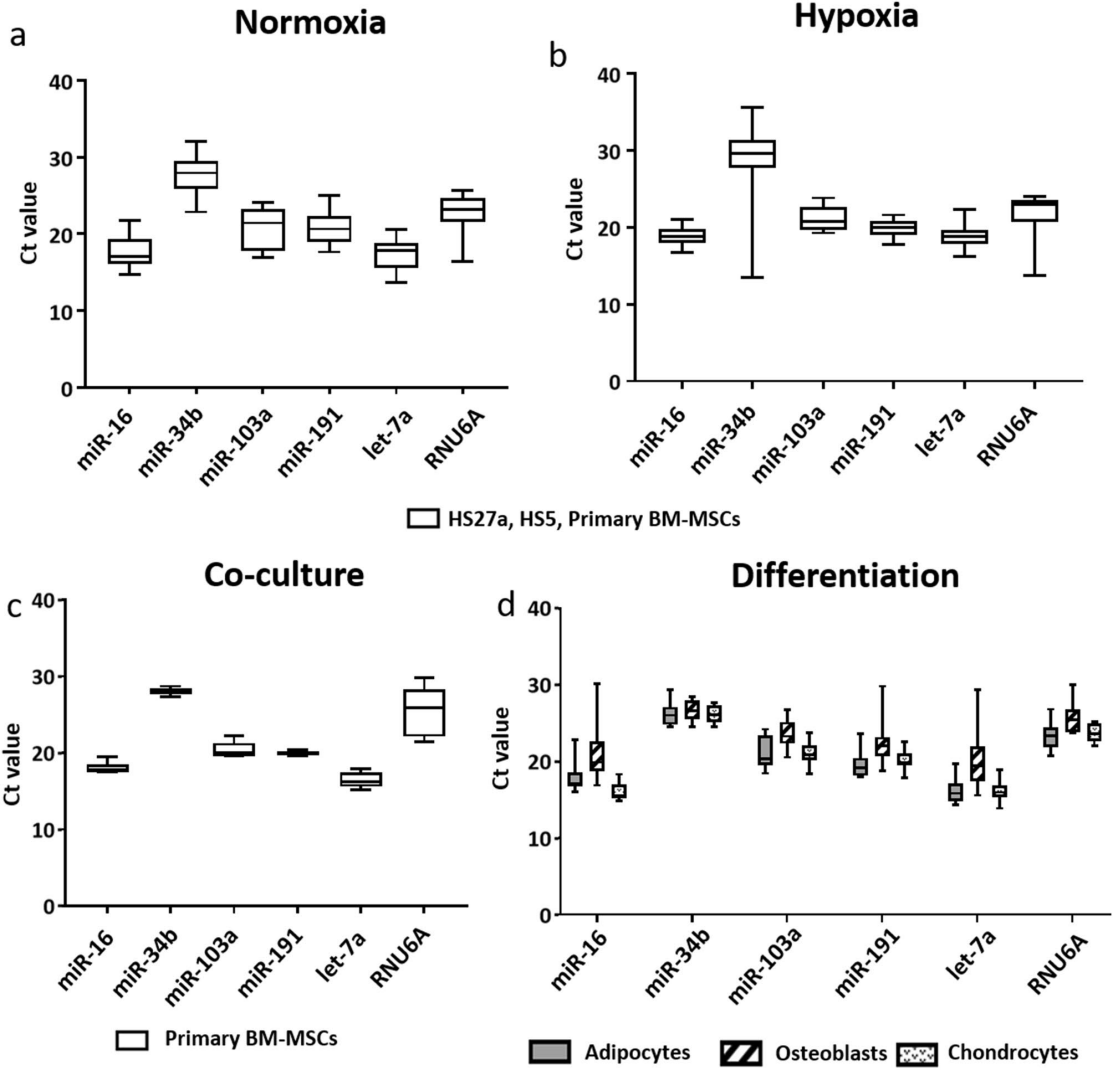
Expression of candidate reference genes in all samples. Determination of Ct values for 6 candidates reference genes tested in HS27a, HS5 and primary BM-MSCs cultivated in normoxia (**a**), hypoxia (**b**), in primary BM-MSCs after 5 days in culture with KG1a cell lines (**c**) and primary BM-MSCs after differentiation in adipocytes, osteoblasts and chondrocytes (**d**).

The expression levels of the RG candidates were measured by RT-qPCR in 8 samples of HS27a, 8 samples of HS5 and 8 samples of primary BM-MSCs (Fig. 2). The Ct value shows that all selected miRs are expressed in all samples whatever the experiment, and within the same range (Ct value between 14 and 30), allowing to study their stability.

### Selection of RGs

In all experiments, results were analysed the same way. First, Ct values were analysed by geNorm to rank RG candidates by stability and determine the optimal number of RGs to use. Next, RefFinder was used to confront geNorm results and determine the Recommended Comprehensive Ranking (RCR). The combination of RCR and optimal number of RGs provides the best RGs to use for each condition.

#### Selection of most stable RGs in BM-MSCs cultured in normoxia and hypoxia

**Figure 3:**
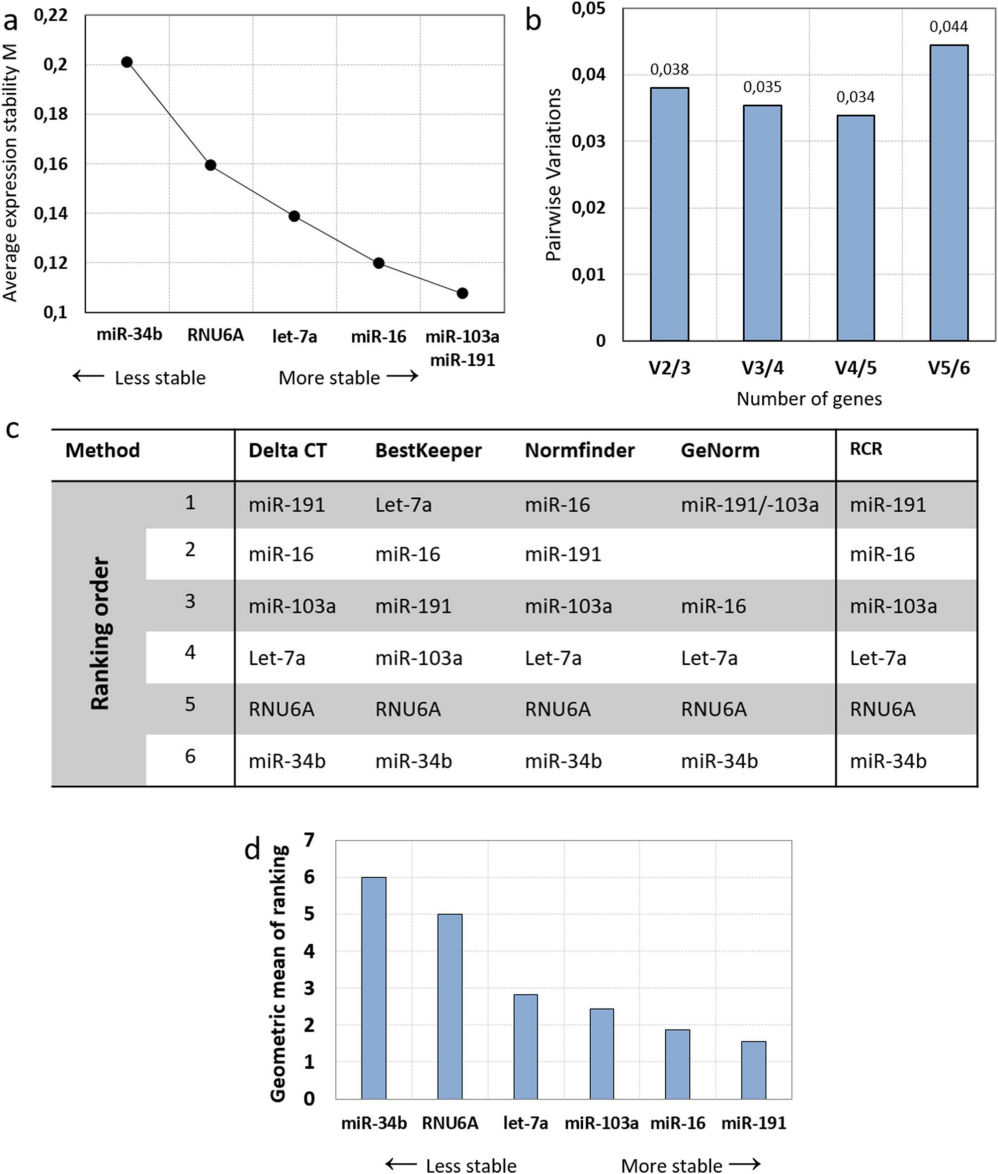
Evaluation of reference genes across normoxia/hypoxia samples. (**a**) Average expression stability values of reference candidate genes by geNorm. Candidates are ranked by stability form less stable to more stable. (**b**) Determination of the optimal number of reference genes for normalization by geNorm. Every bar represents a change in normalization accuracy with the stepwise addition of more reference genes. (**c**) RefFinder analysis of the candidate’s stability. RCR (Recommended Comprehensive Ranking) is the final ranking based on the ranking of each method. (**d**) RCR values of reference genes by RefFinder. Reference candidates are ranked by the geometric mean of the ranking (RCR) obtained by RefFinder.

First, 8 primary mesenchymal stromal cells, HS27a and HS5 cells (8 times each) were cultivated in normoxic and hypoxic conditions. GeNorm was used to calculate the stability using the average pairwise variation. According to the M-value, the most and least stable miRNA were miR-103a-3p/miR-191-5p and miR-34b-3p respectively (Fig. 3a).

geNorm software computed a V_2/3_-value of less than 0.15 indicating that only two miR are needed to ensure a reliable normalization (Fig. 3b). However, as all normalization factors were below 0.15, up to 6 genes could be used as RGs. According to geNorm, the best RG combination was miR-103a and miR-191.

Consolidation of the analysis was achieved thanks to RefFinder, which integrates the normalization determination algorithm of four tools: geNorm, BestKeeper, NormFinder and DeltaCt, calculates the geometric means and ranks the RCRs. Because of their different calculation methods, the ranking obtained by each algorithm is different as seen in Fig. 3c. However, in all results, RNU6A and miR-34b-3p were identified as the less stable miRs. The ranking of the 4 other miRs depends on the algorithm but 3 out of 4 ranked let-7a-5p in 4th position (Fig. 3c). The final RCR are slightly different to the ranking obtained with geNorm with, from less to most stable: miR-34b-3p, RNU6A, let-7a-5p, miR-103a-3p, miR-16-5p, miR-191-5p (Fig. 3d). Because the RCR includes 4 different calculation method, it was decided to be the final stability ranking in our analysis. According to these analyses, miR-191-5p and miR-16-5p is the best normalization combination of RGs in BM-MSCs in normoxic/hypoxic conditions.

#### Selection of most stable RGs in BM-MSCs grown in co-culture with KG1a cells

**Figure 4:**
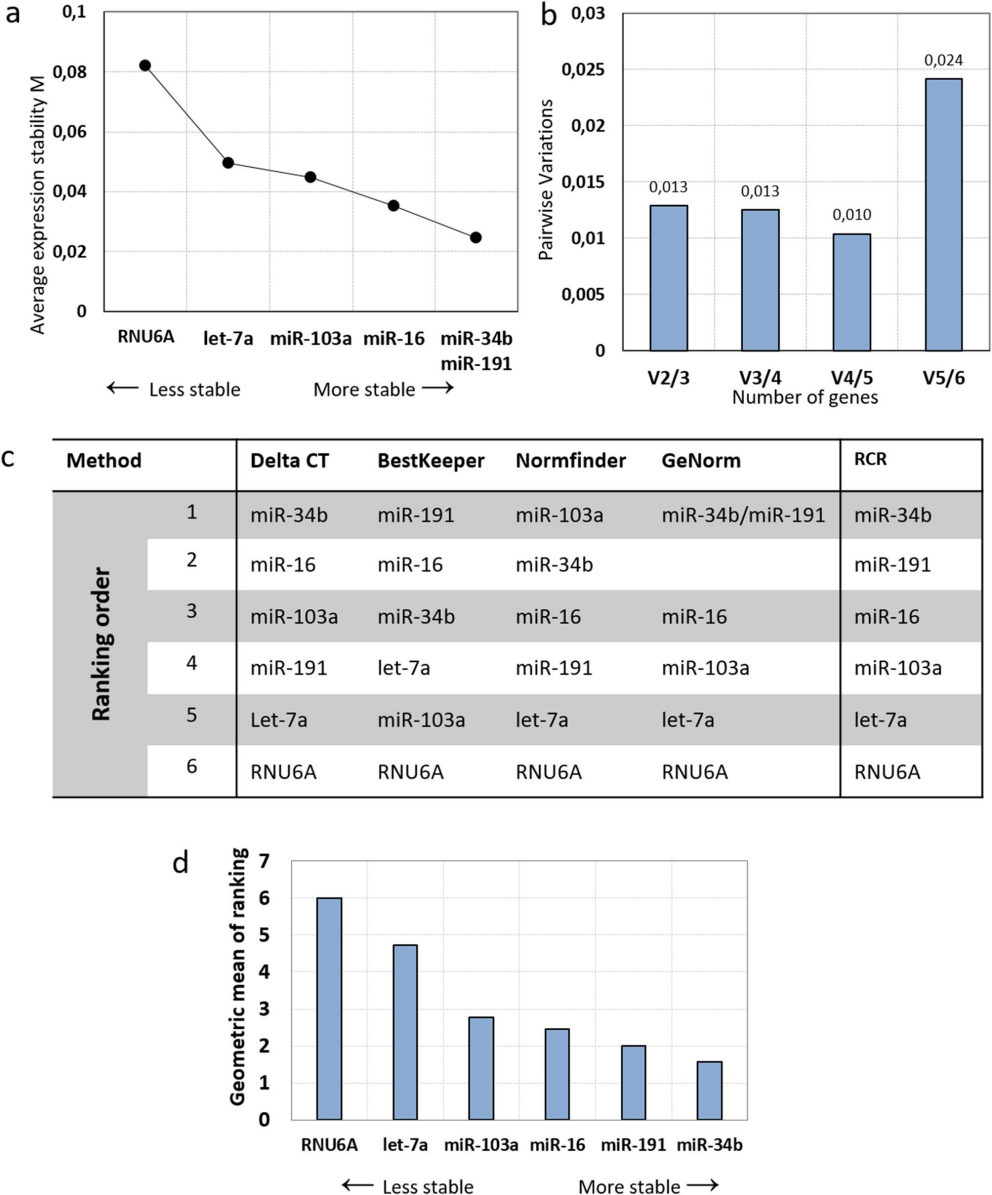
Evaluation of reference genes across co-cultivated samples. (**a**) Average expression stability values of reference candidate genes by geNorm. Candidates are ranked by stability form less stable to more stable. (**b**) Determination of the optimal number of reference genes for normalization by geNorm. Each bar represents a change in normalization accuracy with the stepwise addition of more reference genes. (**c**) RefFinder analysis of the candidate’s stability. RCR (Recommended Comprehensive Ranking) is the final ranking based on the ranking of each method. (**d**) RCR values of reference genes by RefFinder. Reference candidates are ranked by the geometric mean of the ranking (RCR) obtained by RefFinder.

Eight primary BM-MSCs from eight different donors were co-cultivated together with KG1a cells. In these conditions, genes were ranked by geNorm from less stable to most (RNU6A, let-7a-5p, miR-103a-3p, miR-16-5p and miR-34b-3p/miR-191-5p respectively) as shown in Fig. 4a. The normalization factor calculated by geNorm for V_2/3_ and V_3/4_ is 0,013 which set the best number of references at 2 or 3 genes (Fig. 4b). The final RCR set by RefFinder is the same as the ranking obtained by geNorm (Fig. 4c). All the 4 algorithms rank RNU6A as the less stable RG. The final ranking from less stable to most stable is: RNU6A, let-7a-5p, miR-103a-3p, miR-16-5p, miR-34b-3p and miR-191-5p (Fig. 4d). According to these results, miR-34b-3p and miR-191-5p is the best combination of RGs for normalization in BM-MSCs in co-culture.

#### Selection of most stable RGs in BM-MSCs after differentiation

**Figure 5:**
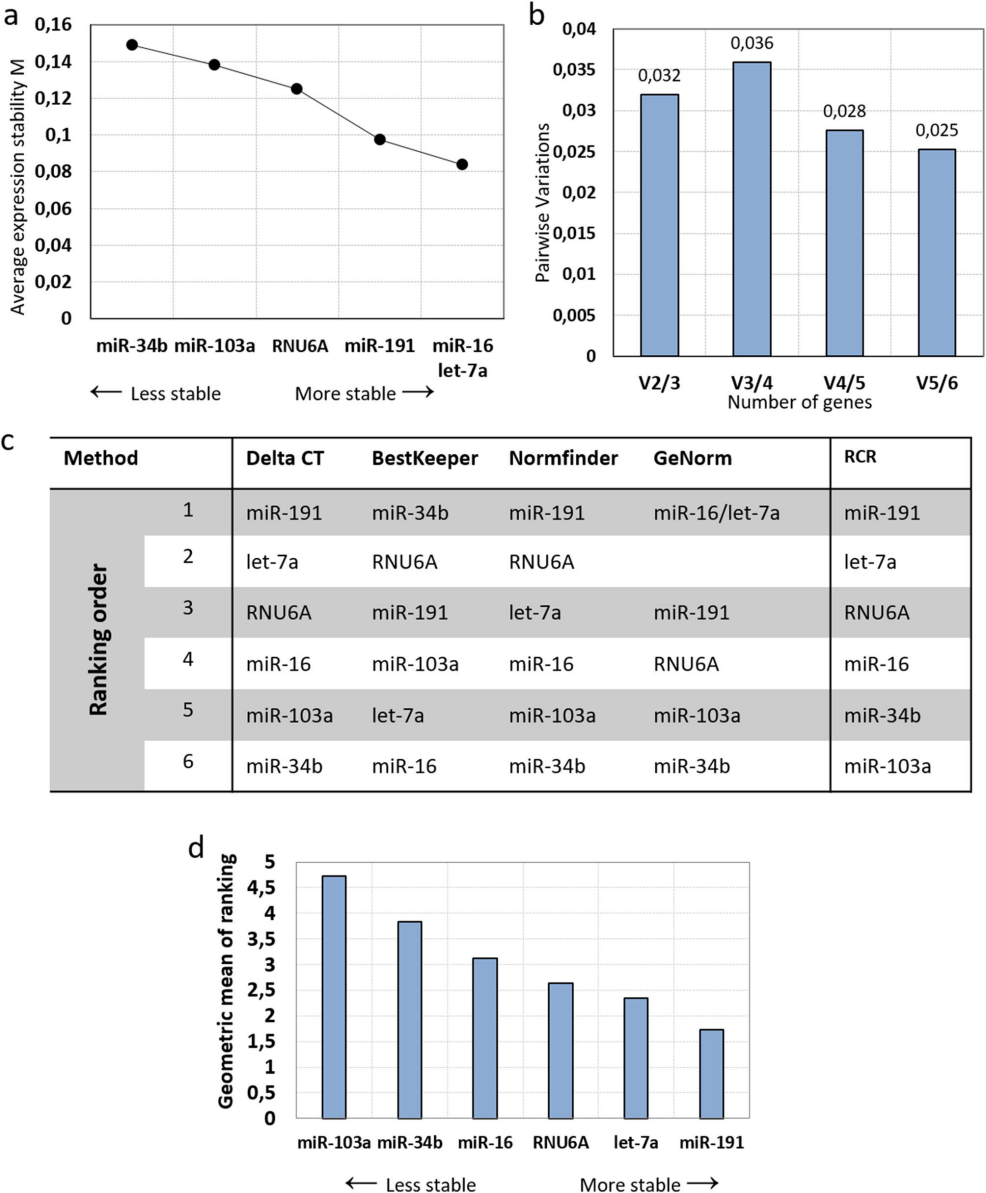
Evaluation of reference genes across differentiated samples. (**a**) Average expression stability values of reference candidate genes by geNorm. Candidates are ranked by stability form less stable to more stable. (**b**) Determination of the optimal number of reference genes for normalization by geNorm. Every bar represents a change in normalization accuracy with the stepwise addition of more reference genes. (**c**) RefFinder analysis of the candidate’s stability. RCR (Recommended Comprehensive Ranking) is the final ranking based on the ranking of each method. (**d**) RCR values of reference genes by RefFinder. Reference candidates are ranked by the geometric mean of the ranking (RCR) obtained by RefFinder.

The 8 primary BM-MSCSs were fully differentiated into chondrocytes, adipocytes or osteoblasts (Supp Data 1). In these conditions, miR-16-5p/let-7a-5p was identified as the most stable by geNorm (Fig. 5a). The best number of RG is two (V_2/3_ = 0,032, Fig. 5b). As shown in Fig. 5c, miR-34b is the less stable for all algorithms but BestKeeper followed by miR-103a-3p. The RCR was slightly different from the ranking obtained with geNorm (Fig. 5d). According to these analyses, miR-191-5p together with let-7a-5p is the best combination of RGs for normalization in differentiated BM-MSCs.

To summarize, the normalization factor of each geNorm analysis confirms that only two RGs are necessary and sufficient for an accurate normalization. miR-191 is always one of the two most stable RGs and it is the only common miR between the three conditions (Table 1). The stability study associates miR-16, miR-34b and let-7a respectively in hypoxia/normoxia, co-culture and differentiated-BM-MSCs.

**Table 1:** Summary of stability analysis results.

BM-MSCs, HS27a, HS5 RGS	Normoxia/hypoxia	Co-culture	Differentiated-BM-MSCs
Most stable	miR-191 and miR-16	miR-191 and miR-34b	miR-191 and Let-7a
Less stable	miR-34b and RNU6A	Let-7a and RNU6A	miR-103a and miR-34b

### Impact of the choice of RGs on miRNA expression quantification

**Figure 6:**
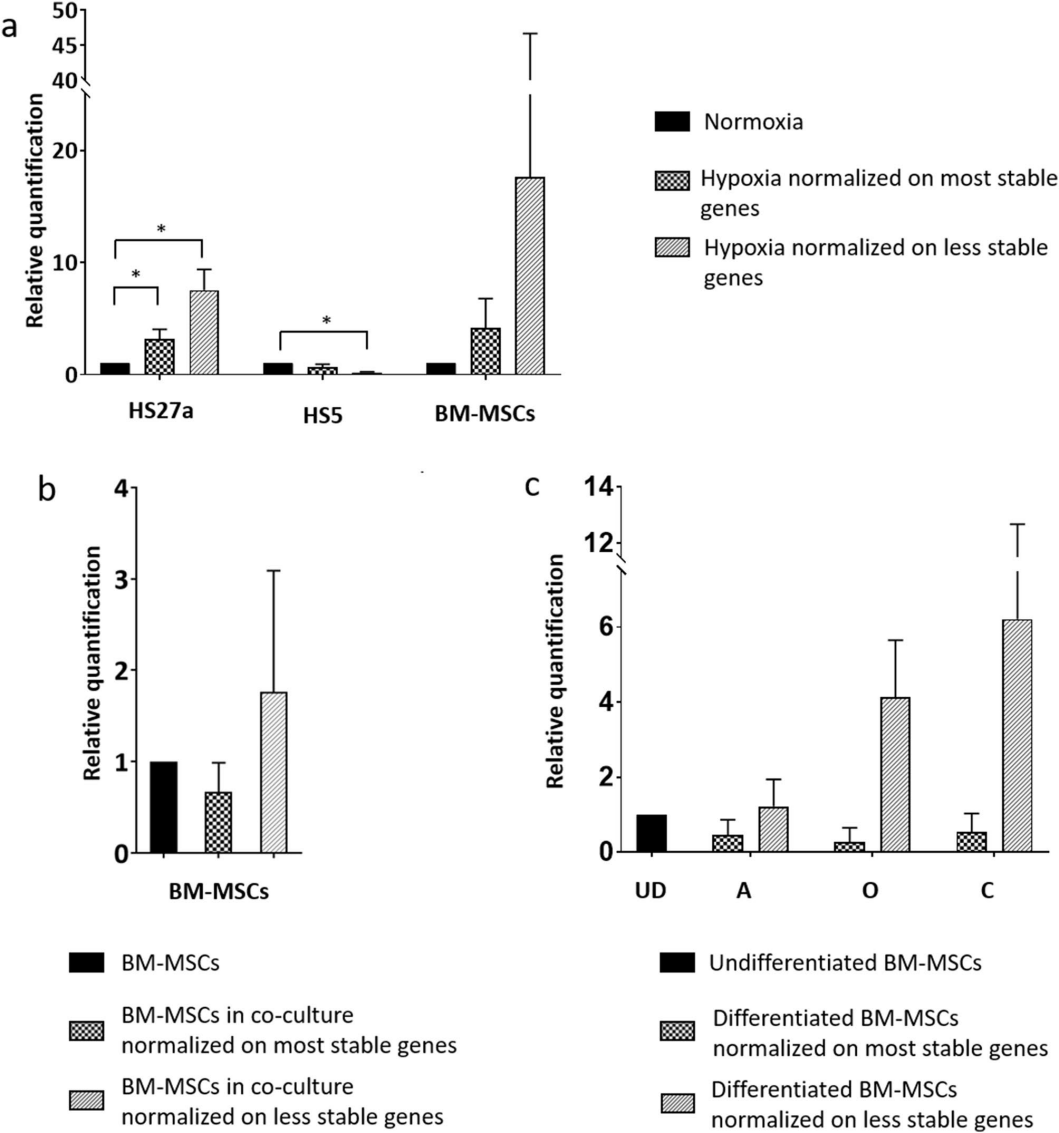
Relative quantification of miR-21-5p in all conditions. For each condition, two references genes have been chosen between the two most stable and the two less stable as previously described. (**a**) Relative quantification of miR-21 in normoxia and hypoxia condition. Most stable reference genes: miR-16/miR-191, less stable reference genes: miR-34b/RNU6A. Normoxia condition has been chosen as reference condition for normalization. (**b**) Relative quantification of miR-21 in co-culture condition. Most stable reference genes: miR-34b/miR-191, less stable reference genes: let-7a/RNU6A. BM-MSCs cultivated alone has been chosen as reference condition for normalization. (**c**) Relative quantification of miR-21 in differentiated experiments with most stable reference genes: let-7a/miR-191 and less stable reference genes: miR-103a/miR-34b. Undifferentiated BM-MSCs has been chosen as reference condition for normalization. *P < 0.05.

The meaning of using a validated reference versus an unvalidated reference on miRNA expression was evaluated in the three experiments. The expression of miR-21-5p was evaluated in HS27a, HS5 cells and primary BM-MSCs in normoxia and hypoxia and on primary BM-MSCs in co-culture and on differentiated primary BM-MSCs. In Fig. 6a, the use of these two combinations affects the statistical significance of the result. In HS27a cells, the significance threshold isn’t affected the relative quantity (RQ) of miR-21-5p is increased with the less stable combination so the fold change is overestimated. In the HS5 cells, it seems that there is a significant down regulation of miR-21-5p expression when using the less stable RGs while with the most stable RGs, there is no significant difference. This result is considered a false positive and leads to a wrong interpretation. For primary BM-MSC samples, there is no statistical difference between the two normalizations although there is a variation between the two RQ and a bigger SD that demonstrates less reliable results. In Fig. 6b and c, the two normalizations are not significantly different, yet opposite tendencies can be observed. High variability was observed upon usage of miR-34b and RNU6A under normoxic and hypoxic conditions of, let-7a and RNU6A under conditions of co-culture and miR-103a and miR-34b under differentiated conditions.

## Discussion

Despite a rapid increase in the use of high-throughput deep sequencing of the miRNA-transcriptome, RT-qPCR still remains the gold standard of expression studies^25–27^. But to perform an appropriate quantification, choosing the right controls for data normalization. This is a key factor for the validation of experimental results. Similarly, choosing valid RGs is a critical step in miRNA expression studies. These candidates must meet several criteria. Ideally, they should be expressed in the target cells and undergo no variations in expression levels nor over time nor in any experimental condition. Thus, finding universal reference miRNA is impossible, since they are expressed in a tissue-specific fashion. A surrogate to universal RG is to normalize thanks to spike-in references. Spike-in reference are synthetic miRNAs that are not present in human miRNAs and behave as exogenous RGs. *C. elegans* miRNAs, cel-miR-39 and cel-miR-34, are commonly used^28,29^. They are added during the extraction of RNA or the RT step^30^. Spike-in references reduces the intra-, but not the inter-experiment variability, the latter depending on culture conditions, treatment etc.… Due to their artificial origin, their behaviour cannot be identical to endogenous miRNAs and thereby makes them a biased control. To perform unbiased normalization, RGs must be endogenous and similar in size and structure as compared to the studied molecules. These constraints rule out mRNAs (average length 1500 nt) and small nuclear and nucleolar transcripts (average length between 60 and 200 nt)^31–33^. As universal endogenous miRNA controls are unavailable, each experiment must be individually set up to find optimal RGs in pilot experiments, in which sample collection and processing must be standardized. This means evaluating the stability and number of candidate miRNAs. The idea to use multiple algorithms to find a reference gene was previously described^34,35^ but never established for miRNAs in our conditions (normoxia/hypoxia, co-culture and differentiation) in BM-MSCs. We follow the normalization process as described by Marabita et al*.* and Drobna et al*.* for the first time in BM-MSCs and BM-MSC-like with 6 selected candidates; miR-16-5p, miR-34b-3p, miR-103a-3p, miR-191-5p, let-7a-5p and RNU6A. Two statistical analysis tools (geNorm and RefFinder) were used. The results shown that among all, miR-191-5p is a safe, conservative choice that can be readily adopted as a standard for internal miRNA studies in BM-MSCs and related cells. Our studies specified that combinations of no more than two miRNAs are necessary and sufficient. It has the advantage of not adding unnecessary expenses to the experiment and ensure an accurate normalization taking into account the innate variability of one single RG^36,37^. As a result, we propose to use miR-191-5p in combination with miR-16-5p in normoxia/hypoxia BM-MSCs culture, together with miR-34b-3p in co-culture or let-7a-5p in differentiation assays. miR-191 has already been described as a suitable RG in several diseases, such as hepatocellular carcinoma (HCC), colorectal adenocarcinoma, and breast cancer or in serum’s exosomal fractions of patients with Hepatitis B or HCC^38–40^. Physiologically, miR-191-5p is described to play a part in essential pathways by targeting numerous targets, such as MDM4 in apoptosis pathway, BDNF3 in proliferation or CDK6 and CCND2 in cell cycle regulation^41^. Previous studies also associated expression changes of miR-191-5p with oncogenic processes. Its upregulation has been observed in several cancers as glioblastoma, HCC, non-small cell lung carcinoma, osteosarcoma, prostate cancer, etc.…^42–46^. In haematology, miR-191-5p is overexpressed in poor prognostic acute myeloid leukaemia^47^. miR-16 is the most frequently used as RG in several models and in circulating miRNA analysis^48–55^ but always with another miR. miR-16 is the most frequently used as RG in several models and in circulating miRNA analyses, always combined with another miR. However, miR-16 is also modified in several conditions such as osteoclastic differentiation^52^ which explain the poor ranking obtained in stability studies in the context of differentiated BM-MSCs. So far, no studies ever identified miR-34b-3p as a candidate RG. miR-34b-3p is considered as a tumour suppressor, which is downregulated in nasopharyngeal carcinoma, bladder and cervical cancer^53–55^. miR-34b-3p is also identified as osteomiR^56^, giving a hint as to why it is an unsuitable RGs in differentiated BM-MSCs. Let-7a-5p belongs to the let-7 family, which is known to be highly express in embryogenesis and to play a tumour suppressor role. It has been validated as RG in lymph node tissues^57^. Notably, let-7a is downregulated in breast cancer, prostate cancer, cervical cancer^58–61^… So far, there is no evidence in the literature of the implication of miR-191-5p, miR-34b-3p and let-7a-5p in the physiology of MSCs. To validate the selected RGs, the relative expression of miR-21-5p was measured by RT-qPCR. As already described in literature^62^, it allowed us to confirm that using unsuitable RGs leads to biased results. Thus, selecting suitable RGs is crucial. Recently, a study showed misuse of RGs for miRNA quantitation in many diseases. As summarized by Madadi et al. in Table 2^63^, various non-coding RNAs have been used in different studies despite the lack of evidence of their eligibility as data normalizers. The authors described, for example, the wide use of RNU6 as RG in many models while stability studies have demonstrated it to be irrelevant in these models. Although, the manuscript of Maldadi et al. did not address the question of BM-MSCs, it drew particular attention to the fundamental need to perform extensive evaluation of RG in miRNA expression studies.

**Table 2:** miR Accession number and reference of primers.

miRNA	Accession number MiRBase/NCBI	Primer reference (Exiqon)
miR-16-5p	MIMAT0000069	YP00205702
miR-34b-3p	MIMAT0004676	YP00204005
miR-103a-3p	MIMAT0000101	YP00204063
miR-191-5p	MIMAT0000440	YP00204306
Let-7a-5p	MIMAT0000062	YP00205727
miR-21-5p	MIMAT0000076	YP00204230
RNU6A (U6snRNA)	NR_004394.1	YP00203907

In conclusion, even though miR-16-5p and miR-34b-3p are involved in osteoblastic and osteoclastic differentiation, hampering their use as universal RGs. We describe the use of miR-191-5p as RG in BM-MSCs under various in vitro culture conditions, note it seems essential to verify this in an in vivo context. We thus recommend the use of the following RG combinations depending on the type of assay: miR-16-5p and miR-191-5p in normoxia/hypoxia experiment, miR-34b and miR-191-5p in co-culture and miR-191-5p and let-7a-5p in differentiated BM-MSCs experiments. This work proposes a panel of RGs, for BM-MSCs or model cell lines in various fields of application such as hypoxia studies, hematopoietic niche modelling or tissue engineering.

## Material and methods

### Primary cells, cell lines and culture

HS27a, HS5 and KG1a cell lines were purchased from the American Type Culture Collection (ATCC). To allow the co-culture with hematopoietic cell lines, HS27a and HS5 were adapted to RPMI media. All cell lines were cultured in RPMI 1640 culture medium (Gibco) supplemented with 10% foetal bovine serum (Thermo Scientific), 20 µM l-glutamine (Gibco), 100 U/ml Penicillin and 100 pg/ml Streptomycin (Gibco). 8 primary BM-MSCs were obtained by iliac crest aspiration from healthy donors (without haematological disorder) undergoing orthopaedic surgery at the University Hospital of Tours, after informed consent, for cell banking according to the Declaration of Helsinki, as approved by the French Ministry of Education and Research (authorization number No. DC-2008-308), isolated and cultured as described^64^. BM-MSCs were used at passage 2 for all experiments. BM-MSCs were cultured in αMEM supplemented with 10% foetal bovine serum (Thermo Scientific), 20 µM l-glutamine (Gibco), 100 U/ml Penicillin and 100 pg/ml Streptomycin (Gibco) and Fibroblast Growth Factor 2 (FGF2). HS27a/HS5 cells and BM-MSCs from donors were cultured at 37 °C with 5% CO_2_. In hypoxic conditions, cells were plated in T-150 flasks at 2.10^6^ cells/flask and cultured in hypoxia chamber 1% O_2_ at 37 °C for 3 days. When cells reached approximately 90% confluence, they were collected and counted.

### Short-term co-culture

5 days short-term co-cultures were performed with confluent stromal layers of primary BM-MSCs and KG1a cells. Five days before co-culture, BM-MSCs were harvested and plated in T-150 flasks at 10,000 cells/cm^2^ in αMEM complete medium. One day before d0, medium was replaced by αMEM without FGF2. On d0, KG1a cells were added at 15,000 cells/cm^2^ in the 24 h. Co-cultures were grown at 37 °C with 5% CO_2_ for 5 days. At d5, adherent fractions were detached with EDTA/Trypsin, washed two times with PBS and FACS sorted. Briefly, after two PBS-wash, cells were incubated with anti-CD45-FITC (Clone HI30, BD Biosciences) and anti-CD90-PE (Clone 5E10, BD Biosciences) into PBS-0.1% BSA-2 mM EDTA buffer during 20 min at 4 °C. Cells were next washed with PBS/BSA/EDTA buffer and sorted with FACS Melody (BD Biosciences). 8 BM-MSCs CD90 + samples were counted and studied.

### In vitro cell differentiation

All differentiation processes were performed on 8 BM-MSCs from 8 different donors.

#### Osteogenic differentiation

When BM-MSCs were 70–80% confluent, growth medium was changed to DMEM high-glucose (4.5 g/l) (Gibco) supplemented with 10% foetal bovin serum, 3 mM NaH_2_PO4 (Sigma-Aldrich), 0,17 mM l-ascorbic acid (Sigma-Aldrich) and 10 mM dexamethasone (Sigma-Aldrich) during 3 weeks. At the end of the differentiation step, cells were fixed in 4% paraformaldehyde and bone nodules were stained with 2% alizarin red (Sigma-Aldrich) or harvested to extract RNA.

#### Adipogenic differentiation

When BM-MSCs were 70–80% confluent, they were cultured in DMEM low glucose (1 g/l) supplemented with 20% FBS, 0.5 mM 3-isobutyl-1-methylxanthine (Invitrogen), 60 µM indomethacin (Sigma-Aldrich) and 1 µM dexamethasone (Sigma-Aldrich) for 2 weeks. At the end of the differentiation, cells were fixed in 4% and lipid droplets were stained with nil red at 1 µg/ml (Sigma-Aldrich) or harvested to extract RNA.

#### Chondrogenic differentiation

Chondrocytes were obtained in micropellet (2.5 × 10^5^ cells/pellet) incubated for 2 to 3 weeks in DMEM high glucose medium (4.5 g/l) supplemented with 10 ng/ml TGF-β1 (Eurobio), 0.1 mM dexamethasone (Sigma-Aldrich), Sodium pyruvate 1 mM (Sigma-Aldrich), 0.17 mM Ascorbic acid 2P (Sigma-Aldrich), 0.35 mM proline (Sigma-Aldrich), and Isulin-Transferrin-Selenium 1x (Cambrex). Aggregates were fixed in 4% paraformaldehyde and include in paraffin. Glycosaminiglycans were stain with 3% alcian blue (Sigma-Aldrich) or harvested to extract RNA.

### RNA extraction

Total RNA from BM-MSCs and cell lines was prepared using NucleoSpin miRNA kit (MACHEREY–NAGEL) according to manufacturer’s instructions. Total RNA from adipocytes and osteoblasts was prepared using TRIzol Reagent (Invitrogen) per manufacturer’s instructions respectively after 2 and 3 weeks of culture in the differentiation media. Total RNA from chondrocytes was prepared using TRIzol Reagent (Invitrogen) after several freezing/thawing cycles as described by Solchaga et al.^65^ after 2 to 3 weeks of culture in the differentiation media. The RNA concentration was then determined with the NanoDrop Lite (Thermo Scientific).

### Candidate reference genes and primer

As suggested by Andersen et al*.*^66^ , a minimum of 5 candidates reference genes should be analysed. miR-16-5p, miR-103a-3p and miR-191-5p were chosen because of their reported expression in BM-MSCs^23^ or their use in stability analysis in other models^67–70^. miR-21-5p was chosen for validation experiment since it is described to be expressed in BM-MSCs and could be used as BM-MSC marker^23^. The corresponding accession numbers of the candidate selected genes and miR-21-5p are listed in Table 2.

### Reverse transcription and quantitative real time PCR

200 ng of each total RNA sample were reverse transcribed with miRCURY LNA RT Kit (Qiagen) according to manufacturer’s instructions. For qPCR, miRCURY LNA SYBR Green PCR Kit (Qiagen) was used according to manufacturer’s instructions with a LightCycler 480 (Roche). All primers were obtained from Qiagen.

### Data analysis

To determine the best combination of reference gene among the selected candidates, the stability of expression of candidate genes was determined using several statistic tools. NormFinder calculates the stability of expression of the candidate reference genes taking account of the inter- and intragroup variations. The stability value depends of these two analyses and of the transcription variation of the candidate reference gene. The lowest the stability value is, the more stable is the candidate gene^66^. GeNorm determine the stability (called M value) with the average pairwise variation between one candidate gene and all other candidate genes. The gene with the highest variation in expression is eliminated and so on until the best pair of reference genes is established^36^. For the optimal number of RG, geNorm calculate the normalization factor V_(n/n+1)_ (where n is the number of RG). For example, the normalisation factor for 2 genes corresponds to V_(2/3)_, for 3 genes to V_(3/4)_…. Vandesompele et al. set a cut off for the normalization factor value at 0.15^36^. If the normalisation factor is below 0.15 than the inclusion of an additional control gene is not required. BestKeeper calculates the standard deviation (SD) and the coefficient of variance (CV) from Ct levels of the candidate genes. The more stable reference genes is the one who possesses the lowest CV and SD^71^. The DeltaCt method is a comparison of the relative expression. The stability of the candidate gene is ranked according the reproducibility of the gene expression in the samples^72^. To obtain a final rank of our candidate reference genes, the geometric mean of ranks was calculated by RefFinder^72^ from the ranks observed from each program. Boxplots, histograms and statistical analysis were performed using Prism 7 software (GraphPad). The design of this study is depicted in Fig. 1.

## Supplementary information

Supplementary Data.
