# Supplementary material for: The crucial choice of reference genes: identification of miR-191-5p for normalization of miRNAs expression in bone marrow mesenchymal stromal cell and HS27a/HS5 cell lines
Source: Sci Rep. 2020 Oct 20;10:17728. doi: 10.1038/s41598-020-74685-7 (PMC7576785; doi:10.1038/s41598-020-74685-7)
Supplement: Supplementary file 1 — Supplementary Data. [file 41598_2020_74685_MOESM1_ESM.pdf]

The crucial choice of reference genes: Identification of miR-191-5p for normalization of miRNAs expression in bone marrow mesenchymal stromal cell and HS27a/HS5 cell lines.

É Costé[1], F Rouleux-Bonnin[1*]

[1] CNRS ERL7001 GICC Team LNOx – University of Tours - France
*email: florence.rouleux-bonnin@univ-tours.fr

Supplementary Data 1 & 2

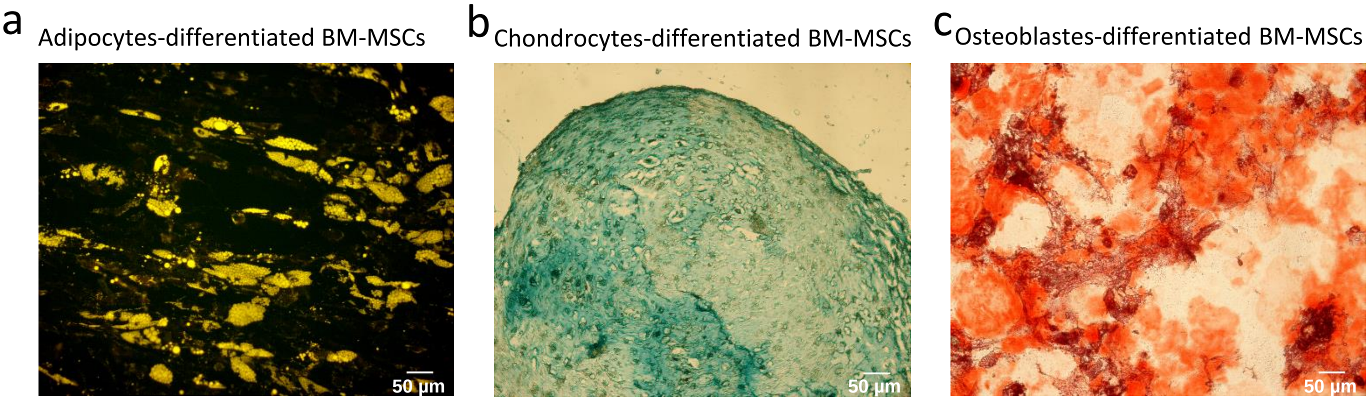

Supplementary Data 1: **Control of BM-MSCs differentiation.** Pictures shown (a) intracellular lipids in nile red in adipocytes, (b) glycoaminoglycans in alcian blue in chondrocytes, (c) calcium deposits in alizarin red in osteoblasts

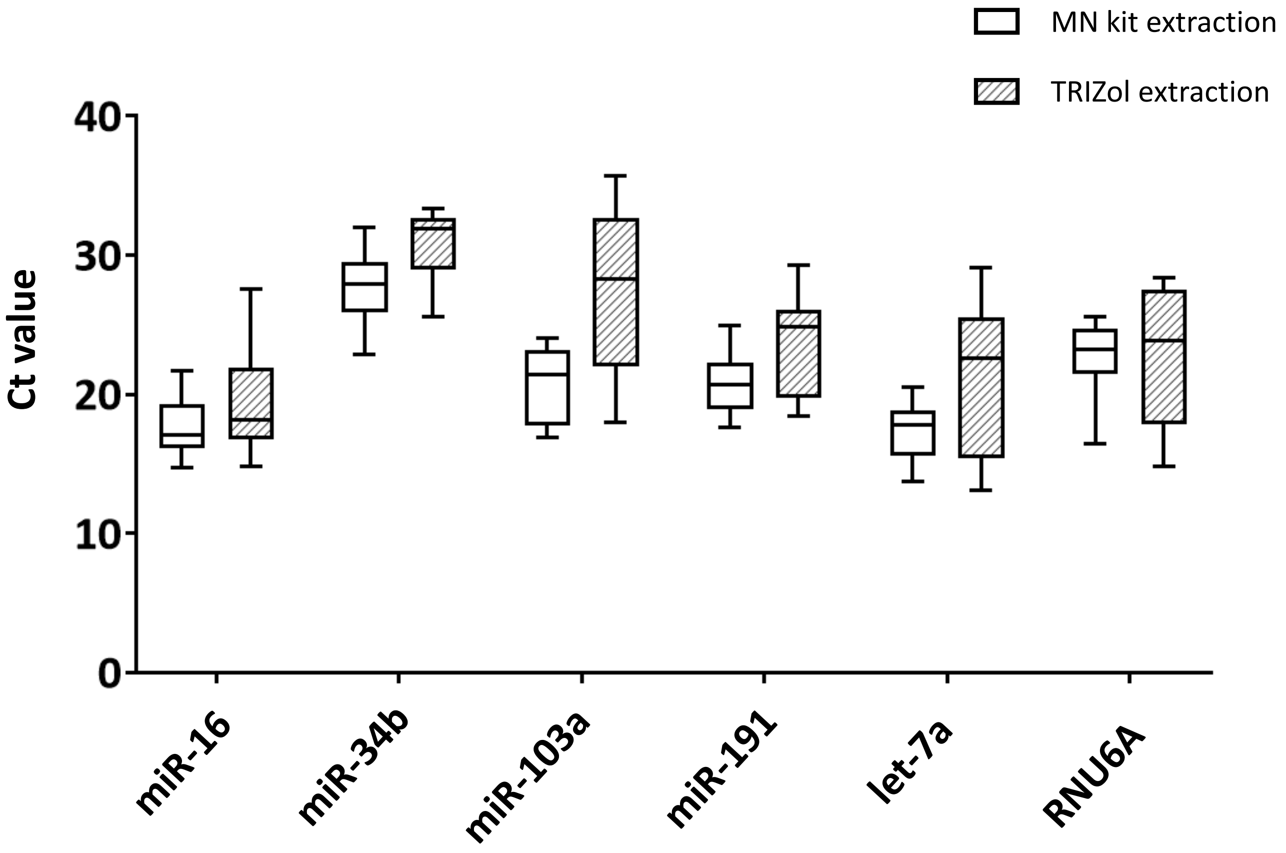

Supplementary data 2: **Expression of candidate reference genes** HS27a, HS5 cells and primary BM-MSCs have been cultivated in normoxia with 8 repetitions each and RNA have been extracted using NucleoSpin miRNA kit (Macherey-nagel) or TRIzol (Invitrogen)
